# Urinary Neutrophil Gelatinase-Associated Lipocalin Is Excellent Predictor of Acute Kidney Injury in Septic Elderly Patients

**DOI:** 10.14336/AD.2017.0307

**Published:** 2018-04-01

**Authors:** Erica Pires da Rocha, Lais Gabriela Yokota, Beatriz Motta Sampaio, Karina Zanchetta Cardoso Eid, Dayana Bitencourt Dias, Fernanda Moreira de Freitas, Andre Luis Balbi, Daniela Ponce

**Affiliations:** University Sao Paulo State-UNESP, Distrito de Rubiao Junior, without number, Botucatu, Sao Paulo, Brazil; University Sao Paulo State-UNESP, Distrito de Rubiao Junior, without number, Botucatu, Sao Paulo, Brazil; University Sao Paulo State-UNESP, Distrito de Rubiao Junior, without number, Botucatu, Sao Paulo, Brazil; University Sao Paulo State-UNESP, Distrito de Rubiao Junior, without number, Botucatu, Sao Paulo, Brazil; University Sao Paulo State-UNESP, Distrito de Rubiao Junior, without number, Botucatu, Sao Paulo, Brazil; University Sao Paulo State-UNESP, Distrito de Rubiao Junior, without number, Botucatu, Sao Paulo, Brazil; University Sao Paulo State-UNESP, Distrito de Rubiao Junior, without number, Botucatu, Sao Paulo, Brazil; University Sao Paulo State-UNESP, Distrito de Rubiao Junior, without number, Botucatu, Sao Paulo, Brazil

**Keywords:** elderly, acute kidney injury, biomarker, NGAL

## Abstract

Elderly is the main age group affected by acute kidney injury (AKI). There are no studies that investigated the predictive properties of urinary (u) NGAL as an AKI marker in septic elderly population. This study aimed to evaluate the efficacy of uNGAL as predictor of AKI diagnosis and prognosis in elderly septic patients admitted to ICUs. We prospectively studied elderly patients with sepsis admitted to ICUs from October 2014 to November 2015. Assessment of renal function was performed daily by serum creatinine and urine output. The level of uNGAL was performed within the first 48 hours of the diagnosis of sepsis (NGAL1) and between 48 and 96 hours (NGAL2). The results were presented using descriptive statistics and area under the receiver operating characteristic curve (AUC-ROC) and *p* value was 5%. Seventy-five patients were included, 47 (62.7%) developed AKI. At logistic regression, chronic kidney disease and low mean blood pressure at admission were identified as factors associated with AKI (OR=0.05, CI=0.01-0.60, *p*=0.045 and OR=0.81, CI=0,13-0.47; *p*=0.047). The uNGAL was excellent predictor of AKI diagnosis (AUC-ROC >0.95, and sensitivity and specificity>0.89), anticipating the AKI diagnosis in 2.1±0.3 days. Factors associated with mortality in the logistic regression were presence of AKI (OR=2.14, CI=1.42-3.98, *p*=0.04), chronic obstructive pulmonary disease (OR = 9.37, CI =1.79-49.1, *p*=0.008) and vasoactive drugs (OR=2.06, CI=0.98-1.02, *p*=0.04). The accuracy of NGALu 1 and 2 as predictors of death was intermediate, with AUC-ROC of 0.61 and 0.62; sensitivity between 0.65 and 0.77 and specificity lower than 0.6. The uNGAL was excellent predictor of AKI in septic elderly patients in ICUs and can anticipate the diagnosis of AKI in 2.1 days.

Acute kidney injury (AKI) is a common and important occurrence in the Intensive Care Units (ICU) [1], and several studies support the statement that the elderly patient and sepsis are at the highest risk for AKI, which are associated with considerable mortality (40%) [2-5].

In the process of ageing, there is loss of muscular mass and therefore, the plasmatic creatinine levels of the elderly can be lower than the normal values, masking an increase in kidney injury pathologies, leading to delayed or late diagnosis, justifying the search for biomarkers of early injury, such as neutrophil gelatinase-associated lipocalin (NGAL) [6-10].

Although considered an early biomarker, NGAL levels can be elevated after activation of neutrophils, suggesting influence of systemic inflammation and infections [11-14].

Studies on pediatric ICU patients have shown pNGAL to be a nonspecific predictor [15] and uNGAL to be a good predictor of AKI [16]. Indeed, Bagshaw et al. [17] in a study that included 83 AKI patients showed that both p- and uNGAL were higher in septic versus nonseptic patients. Martensson et al. [18] performed a study that evaluated 65 septic patients admitted to ICU and showed that pNGAL was not a good predictor of AKI because it was elevated in septic patients without AKI, probably due to the systemic infections. There are no studies have investigated the predictive properties of NGAL as an AKI marker in a septic elderly population. Given the higher mortality rate of elderly patients with sepsis and AKI and lack of studies, we decided to investigate the role of uNGAL as predictor of AKI in septic elderly patients admitted to ICU.

## MATERIALS AND METHODS

### Study Population

This was an observational study. All elderly patients admitted to the general ICU at Botucatu School of Medicine, Sao Paulo, Brazil (University of Sao Paulo State-UNESP) were followed prospectively from the time of admission to ICU through ICU discharge. Nephrology fellows visited the ICU daily from October 2014 to November 2015 and collected data on all elderly patients admitted in this ICU. We included patients 60 years of age or older who had sepsis according to “Survival Sepsis Campaign 2012” [19].

**Figure 1.**
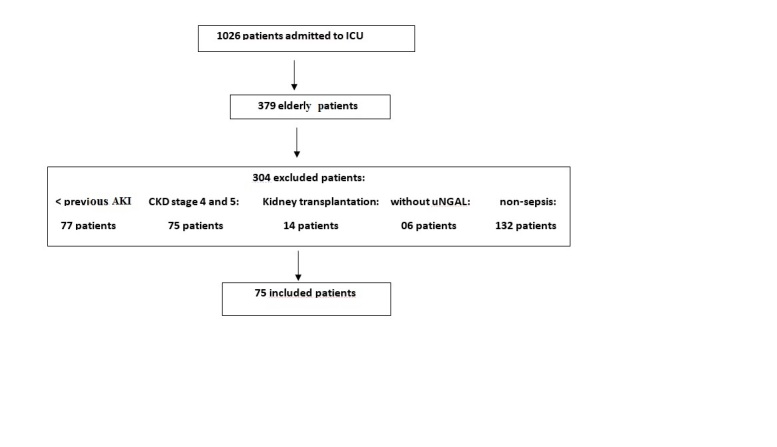
Screening and enrollment.

Elderly was defined according to Brazilian law (> 60 years old) and AKI defined according to the Kidney Disease Improving Global Outcomes (KDIGO) criteria based on increase of serum creatinine or decrease of urine output [20]. We excluded chronic kidney disease (CKD) patients stages 4 and 5 (clearance de creatinine < 30 ml/min), kidney transplantation, ICU stay < 24 h and patients already admitted in ICU with AKI. Baseline glomerular filtration rate was estimated using the modification of the diet in renal disease equation [21]. Baseline creatinine was defined as the lowest serum creatinine value in the last 6 months before AKI or, for those without this measurement, the lowest value achieved during hospitalization in the absence of dialysis [22]. Complete data on inclusions and exclusions are shown in Figure 1.

**Table 1:** Patients demographics and clinical characteristics (n=75).

Characteristics	Septic elderly patients N=75	Non-AKI patients N= 28	AKI patients N= 47	*p*
Age (years)	71.4±7.5	71.7 ±7.6	71.3±7.6	0.82
Male sex n (%)	39 (52)	12 (42,9)	27 (57,4)	0.22
Baseline creatinine	1.16±0.52	0.8±0.3	1.3±0.3	0.0009
Comorbidities n (%)				
Hypertension	45 (60)	15 (53,5)	30 (63.8)	0.38
Diabetes	25 (33.3)	7 (25)	18 (38.3)	0.23
CVC disease	35 (46.7)	11 (39.3)	24 (51.1)	0.32
CKD	34 (49.3)	4 (17.4)	30 (63.8)	0.0002
COPD	17 (22.7)	8 (28.6)	9 (19.1)	0.34
Mechanical ventilation n (%)	49 (65.3)	16 (57.1)	33 (70.2)	0.25
Noradrenaline use n (%)	59 (78.7)	19 (67.9)	40 (85.1)	0.07
Infection Focus n (%):				0.36
Lung	39 (52)	12(42.8)	27 (57.4)	
Urine	18 (24)	10 (35.7)	8 (17.0)	
abdominal	7 (9,3)	2 (7.1)	5 (10.6)	
Urine output in 24h (ml)	874.2±729.7	797.8±535.6	919.2±826.4	0.52
APACHE II	17.7±6.8	14±4.8	19.4±7.0	0.0045
Temperature	36.8±1.1	36.4±0.9	37.1±1.1	0.007
Outcome n (%):				0.03
discharge	35 (46.7)	18 (64.2)	17 (36.2)	
death	40 (53.3)	10 (35.7)	30 (63.8)	

Values expressed as mean and standard deviation or median and interquartile range AKI- acute kidney injury, MBP- Mean Blood Pressure, CVC- cardiovascular disease, CKD- chronic kidney disease, COPD: chronic obstructive pulmonary disease

Variables previously reported to be associated with AKI and death in other populations were included in the risk factor analysis. Baseline data, including demographics, medical history, and severity, were collected prospectively on each patient by review of the medical record. Key risk factors analyzed are displayed in Table 1. Laboratory characteristics were recorded for the first ICU day and vital signs, hemodynamic and laboratory data were recorded each day during ICU stay. Renal function was assessed daily based on creatinine levels and urine output. Serial Acute Physiology and Chronic Health Evaluation (APACHE) II score was computed on the first ICU day and AKI was classified according to KDIGO stage after 7 days from AKI diagnosis [23].

Urine was analyzed for NGAL and Cr within the first 48 hours after admission (classified as NGAL1), and between 48 and 96 h (NGAL2). The samples were centrifuged and stored at minus 80-degree Celsius and were analyzed subsequently. NGAL was measured by the *enzyme linked immunosorbent assay* (ELISA). Expected normal uNGAL level was less than 2.2 ng/mL. We performed level of uNGAL in 20 healthy subjects between 30 and 50 years old and the mean was 2.2 ± 0.29 ng/mL. Serum and urinary Cr was measured by the enzymatic colorimetric test based on Jaffé reaction (alkaline picrate).

The Ethics Committee of the Botucatu School of Medicine, UNESP, approved this study in March 2015 (protocol number 986.477), with a waiver of informed consent given its observational nature.

### Statistical Analysis

Data analysis was performed using SAS for Windows (version 9.2, SAS Institute, Cary, NC, USA, 2012). Continuous variables with normal distribution were described using means ± standard deviation and those with a non-normal distribution as median and interquartile range. Categorical variables were presented as ?? (%). For the analysis of continuous variables, Student’s ??-test was used for data with a parametric distribution and the Kruskal-Wallis test for non-normal data. For the analysis of categorical variables, a chi-square test was used. Diagnostic characteristics of uNGAL in predicting AKI and death were assessed by calculation of the area under the receiver operating characteristic curve (AUC-ROC). AUCROC analysis was performed by comparing AKI patients with all non-AKI patients and by comparing survivor patients with those non-survivor patients. The optimal cutoff points were determinated by the highest values of sensitivity and specificity showed in AU-ROC analysis. In all tests, differences were considered significant at 5%.

## RESULTS

Seventy-five patients were included in the final analysis (Fig. 1). Mean age was 71.4 ± 7.53 years, 52% were male, most of them had comorbidities (65.4%), and hypertension, chronic kidney disease (CKD), cardiovascular disease, and diabetes mellitus were the most frequent (in 60, 49.3, 46.6, and 33.3% of patients, respectively). APACHE II score was 17.7 ± 6.89 and the need for mechanical ventilation and noradrenalin use in the first 24 hours after admission to ICU was 65.3 and 78.7%, respectively. The main source of infection was the lung (52%), followed by the urinary tract (24%). Forty-seven patients (62.7%) developed AKI and most of patients were classified as KDIGO 3 (43.5%), while KDIGO 1 occurred in 30.4% and KDIGO 2 in 28.1%. ATN-ISS was 0.63±0.25, acute renal replacement therapy was indicated in 5%, and mortality rate was 53.3%.

**Table 2:** Patients demographics and clinical characteristics (n=75) according to outcome.

Characteristics	Non-Survivors (N=40)	Survivors (N=35)	p
Male sex n (%)	22 (55)	17 (48.6)	0.57
Age (years)	70.9±7.26	72±7.9	0.53
MBP	78.4±22.3	89.3±21.8	0.03
Comorbidities n (%)			
Hypertension	24 (60)	21 (60)	1.0
Diabetes	13 (32.5)	12 (34.3)	0.87
Dyslipidemia	6 (15)	10 (28.6)	0.15
Cardiovascular disease	19 (47.5)	16 (45.7)	0.87
Liver disease	4(10)	0	0.05
CKD	19 (47.5)	15 (42.8)	0.71
COPD	12 (34.3)	10 (28.7)	0.02
Baseline creatinine	1.2±0.5	1.0±0.4	0.13
Noradrenaline use n (%)	36 (90)	23 (65.7)	0.01
Mechanical ventilation n (%)	29 (72.5)	20 (57.1)	0.16
Urine output in 24h (ml)	842.6±414.2	912.5±624.3	0.70
Focus n (%):			0.81
lung	20 (50)	19(54.3)	
urine	11(27.5)	7(20)	
APACHE II	19.7±6.6	15.1±6.5	0.009
AKI	28 (70)	15 (42.9)	0.03
KDIGO n (%):			
I	6 (20.7)	8 (53.3)	0.02
II	7 (25)	4 (26.6)	
III	15 (53.6)	3 (20)	
Need for dialysis n (%)	3 (7.5)	2 (5.7)	0.63

Values expressed as mean and standard deviation or median and interquartile range. AKI- acute kidney injury, MBP- Mean Blood Pressure, CKD- chronic kidney disease, COPD: chronic obstructive pulmonary disease

The non-AKI (n=28) and AKI group (n=47) were similar in age, hypertension, diabetes, cardiovascular disease, need for mechanical ventilation and vasoactives drugs. AKI group had higher baseline creatinine (0.8 ± 0.30 vs. 1.3±0.3 *p*=0.0009), higher APACHE II (19.4±7.04 vs 14±4.86, *p*=0.00045), higher CKD (65 vs.17%, *p*=0.0002), lower median blood pressure (MBP) at admission and sepsis was the main diagnosis at ICU admission (48.9 vs. 11.1%) (Table 1). In multivariable regression analysis, independent risk factors for AKI included MBP lower than 65 mmHg at moment of admission (OR=0.81, CI=0.130-0.472, *p*= 0.047) and CKD (OR= 2.01; 95% CI 1.17-2.92; *p*=0.045) (Table 2).

Mortality was higher in AKI patients (70% vs. 42.9%, *p* = 0.03. Table 3 shows the clinical and laboratory characteristics of the population according to the hospital outcome. The groups were similar regarding gender, age, comorbidities, baseline creatinine, temperature at admission, need for mechanical ventilation, noradrenaline use and NGAL values. The two groups were statistically different in MBP at moment of admission to ICU, which was significantly lower in non-survivor patients (78.4±22.3 vs 89.3±21.8, *p*=0.0), in the need for noradrenalin use, APACHE II and in the diagnosis of septic shock at ICU admission, being higher in non-survivor group (90% vs. 65.7%, *p*=0.01; 19.7±6.61vs. 15.1±6.5, *p*=0.009 and 45 vs. 23.5%, *p*=0.4, respectively). Prevalence of AKI and chronic obstructive pulmonary disease (COPD was significantly higher in non-survivor patients (87.5% vs. 45.7%, *p*=0.0001; 34.3 vs 12.5%, *p*=0.025).

**Figure 2.**
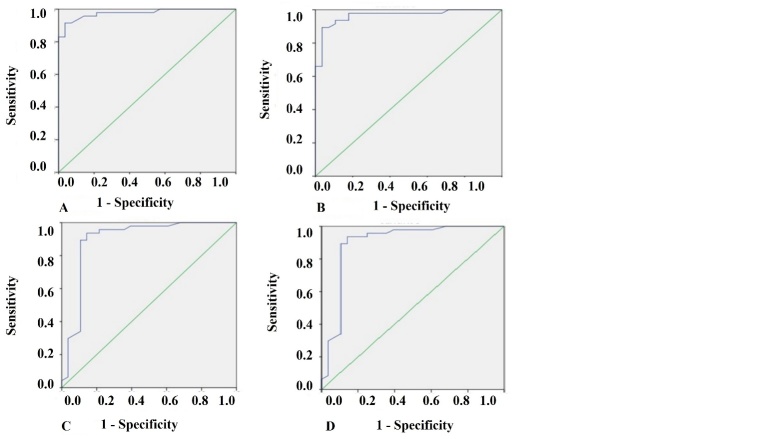
ROC analysis of uNGAL in septic elderly patients with AKI vs non-AKI. A) ROC analysis of uNGAL measured on first 48 hours of admission to ICU in septic elderly patients with AKI vs non-AKI. B) ROC analysis of uNGAL measured between 48-96 hours of admission to ICU in septic elderly patients with AKI vs non-AKI. C) ROC analysis of uNGAL/uCr measured on first 48 hours of admission to ICU in septic elderly patients with AKI vs non-AKI. D) ROC analysis of uNGAL/uCr measured between 48-96 hours of admission to ICU in septic elderly patients with AKI vs non-AKI.

COPD (OR=9.37, 95% CI 1.79 - 49.1, *p*=0.008), AKI (OR=2.14, 95% CI 1.42-3.98, *p*=0.04) and noradrenaline use (OR=2.06, 95% CI=0.98-1.02, *p*=0.04) were identified as independent risk factors for death in multivariable regression analysis (Table 2).

**Table 3:** Multivariable analysis for AKI and death risk (n=75).

AKI	OR	CI (95%)	*p*
CKD	0.05	(0.01 -0.60)	0.04
Temperature	1.84	(0.34 - 9.81)	0.71
MBP	0.81	(0.13 - 090)	0.04
**Death**	**OR**	**CI (95%)**	***p***
Noradrenaline use	2.06	(1.14 - 1.63)	0.04
Mechanical ventilation	0.38	(0.07- 1.94)	0.24
COPD	9.37	(1.79 - 49.10)	0.008
CKD	0.39	(0.08 - 1.73)	0.21
AKI	2.14	(1.42 - 3.98)	0.04

AKI: acute kidney injury, MBP: mean blood pressure, CKD: chronic kidney disease, COPD: chronic obstructive pulmonary disease.

uNGAL1 and uNGAL2 in AKI group showed higher values than non-AKI group: µ1 NGAL (19.1±4.6 vs 6.8±3.7 µg/ml; *p*<0.0001), u2 NGAL (19.6±5.6 vs. 6.1±4.0 µg/ml; *p*<0.0001), uNGAL/uCr 1 (127.9±30.7 vs 56.6±39.9 µg/mg; *p*<0.0001) and uNGAL/uCr 2 (138.3±31.6 vs 58.8±43.4 µg/mg; *p*<0.0001). Only uNGAL/uCr 1 was higher in nonsurvival group when compared with survival patients (113.9±47.2 vs. 86.9±27.7 ng/mg, *p*=0.0001). The two groups were similar in uNGAL1 and 2 (Table 5).

**Table 4:** Urinary NGAL values according to presence of acute kidney injury.

	Non-AKI N=28	AKI N=47	*p*
**uNGAL (ng/ml)**			
at moment 1 (<48h)^*^	6.8±3.7	19.1±4.6	<0.0001
at moment 2 (48-96h)	6.1±4.0	19.6±5.6	<0.0001
**uNGAL /uCr (ng/mg):**			
at moment 1 (<48h)	56.6±39.9	127.9±30.6	<0.0001
at moment 2 (48-96h)	58.8±43.4	138.3±31.5	<0.0001

Values expressed as median and interquartile range. u-urinary; AKI- acute kidney injury;

* after admission to intensive care unit.

**Table 5:** Urinary NGAL values according to patient outcome.

	Non-survivors N=35	Survivors N=40	*p*
**uNGAL (ng/ml)**			
at moment 1 (<48h^*^)	15.9±7.3	12.9±7.2	0.08
at moment 2 (48-96h)	15.9±8	13.0± 8.3	0.13
**uNGAL /uCr (ng/mg)**			
at moment 1 (<48h)	113.9 ±47.2	86.9±27.7	<0.001
at moment 2 (48-96h)	122.3±50.8	93± 51.7	0.19

Values expressed as median and interquartile range. u-urinary; AKI- acute kidney injury;

* after admission to intensive care unit

Figure 2 display the receiver operator curves (ROC) for uNGAL as predictor of AKI. The areas under the curve for uNGAL1, uNGAL2, uNGAL1/uCr1, and uNGAL2/uCr2 were 0.97, 0.96, 0.89, and 0.89, respectively. Both uNGAL and uNGAL/uCr were excellent predictors of AKI within the next 48 h. The optimal cutoff value of each one of them had sensitivity and specificity around 0.9 (Table 6). uNGAL anticipated AKI diagnose according to KDIGO criteria in 2.1±0.3 days.

**Figure 3.**
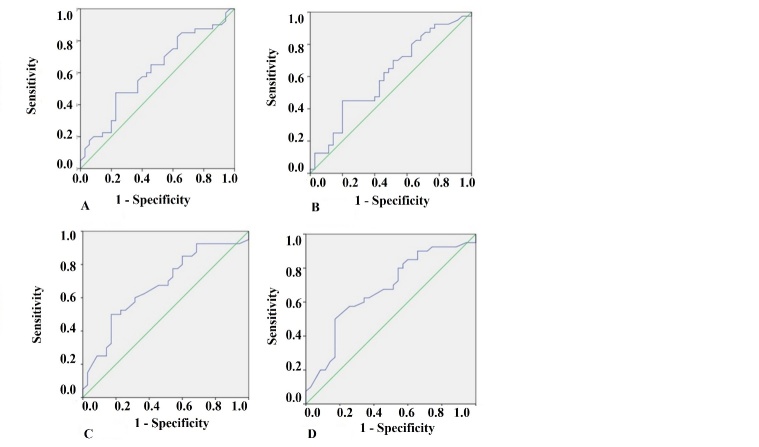
ROC analysis of uNGAL in survivors versus non-survivor’s septic elderly patients. A) ROC analysis of uNGAL measured on first 48 hours of admission to ICU in survivors versus non-survivor’s septic elderly patients. B) ROC analysis of uNGAL measured between 48-96 hours of admission to ICU in survivors versus non-survivor’s septic elderly patients. C) ROC analysis of uNGAL/uCr measured on first 48 hours of admission to ICU in survivors versus non-survivor’s septic elderly patients. D) ROC analysis of uNGAL/uCr measured between 48-96 hours of admission to ICU in survivors versus non-survivor’s septic elderly patients

Concerning uNGAL as predictor of death, the areas under the curve for uNGAL1, uNGAL2, uNGAL1/uCr1, and uNGAL2/uCr2 were 0.61, 0.62, 0.67, and 0.67, respectively (Fig. 3A-D). The optimal cutoff value of each one of them had sensibility between 0.66 and 0.77 and specificity lower than 0.6 (Table 7).

**Table 6:** Urinary NGAL sensitivity and specificity in septic elderly AKI patients (n=47).

	AUC-ROC	*p*	*cutoff*	Sensitivity	Specificity	CI (95%)
**uNGAL1**	*0.97*	0.01	*13.3*	*91.5*	*92.9*	(0.64-0.81)
**uNGAL2**	*0.96*	0.01	*12.7*	*91.5*	*89.7*	(0.55-0.82)
**uNGAL/uCr1**	*0.89*	0.04	*89.9*	*89.4*	*89.6*	(0.68-0.83)
**uNGAL/uCr2**	*0.89*	0.001	*96.7*	*89.4*	*89.7*	(0.63-0.91)

AUC-ROC - receiver operating characteristic curve; Cr - creatinine.

## DISCUSSION

It is widely recognized that the incidence of AKI is increasing over time and is most common in elderly individuals. This is due to many reasons, including elderly are more likely to have renal structural decline and multiple comorbidities [24-27].

Previous studies reported the incidence of AKI among geriatric patients ranged from 22 to 40%, with most patients having stage 1 disease [24, 28,29]. We found that 62.7% of geriatric septic patients had AKI and most of them had AKI stage 3, in disagreement with the literature [25,26]. The in-ICU mortally of geriatric AKI patients (53.3%) was higher than other studies (16-40%) [24, 28-32]. We believe there were differences because these studies also included elderly patients in general wards of the hospital and not only in ICUs.

In accordance with other studies, we observed that the mortality rate of the elderly AKI patients was 63.8% and higher than that of the elderly patients without AKI, which was 35.7%. In a prospective study of elderly AKI patients (>60 years) also performed in Brazil, it was reported that the mortality rate of the elderly AKI patients was 54% [33]. Kohli et al [34] in 2007, in a prospective study found a high mortality rate of 61% in the elderly patients with AKI (age >60 years) in a tertiary care center of India.

**Table 7:** Urinary NGAL sensitivity and specificity in non-survival septic patients (n=35).

	AUC-ROC	*p*	*cutoff*	Sensitivity	Specificity	CI (95%)
**uNGAL1**	*0.61*	0.01	*12.21*	*71.5*	*54.3*	(0.48-0.73)
**uNGAL2**	*0.62*	0.01	*13,.29*	*65.0*	*51.4*	(0.47-0.72)
**uNGAL/uCr1**	*0.67*	0.04	*69.39*	*77.5*	*57.1*	(0.54-0.79)
**uNGAL/uCr2**	*0.67*	0.001	*111.20*	*67.5*	*45.7*	(0.58-0.78)

In our study, AKI, COPD and noradrenaline use were identified as predictors of death in logistic regression analysis. It is similar to previous results reported in the literature. Researchers believed that the high mortality rate in AKI is not explained by the underlying conditions alone. AKI appears to increase the risk of developing severe non-renal complications that lead to death and should not be regarded as a treatable complication of serious illness [32].

In fact, elderly patients are prone to complicating sepsis, AKI and other diseases. And because of the old age, elderly septic AKI patients are easy to develop multiple organs dysfunction system (MODS) which will increase the mortality rate. Therefore, if is important to pay more attention to the treatment of sepsis and concomitant diseases of elderly AKI patients and prevent them from developing dysfunctions of other organs.

In addition to the greater susceptibility to AKI, its diagnosis in elderly patients can be difficult or delayed, due to loss of muscle mass, and consequently lower baseline plasmatic creatinine level, masking an increase of its values in kidney injury pathologies, justifying the search for biomarkers of early injury, such as NGAL [6].

This is the first study of septic elderly patients admitted to ICU to undergo prospective evaluation of uNGAL as a biomarker for AKI and death.

NGAL is a protein with a molecular weight of 25 kDa expressed at low concentrations in different tissues and upregulated especially in injured epithelial cells. Because of that, pNGAL concentration can be high in septic patients, even in the absence of AKI. Then, it may be considered a marker of sepsis, as well as an early biomarker of AKI [29], as shown in several clinical studies [7,8, 16, 35-37].

In study of M°artensson et al. [18], pNGAL and uNGAL were evaluated as predictors of AKI, but the ability of pNGAL to predict AKI in patients with septic shock was poor with an AUC-ROC (0.67) compared to the ability of uNGAL with an AUC-ROC (0.86). The uNGAL was a better predictor of AKI in septic patients than pNGAL probably because it was less affected by presence of sepsis. The pNGAL can be high because of its release into the bloodstream by the systemic activation of neutrophils due to sepsis. The physiological function of the uNGAL is unknown; however, it has a role in renal morphogenesis. The proteomic analysis of studies using animal models revealed uNGAL protein as the earliest product after kidney insult [35,36], representing better the kidney damage than the pNGAL.

Similar results were found in pediatric patients [15] also with septic shock in ICU and the AUC-ROC (0.67), shown to be more sensitive predictor than specific. As the proper sepsis activates and increases the release of NGAL from neutrophils, it is questionable whether it can impair the ability to predict AKI.

In the current study, uNGAL in healthy adults was much lower (2.2±0.2 ng/mL) than that in septic patients, showing it is increased in septic patients without AKI.

Both uNGAL and its relation to uCr within 2 and 4 days after admission to ICU were excellent predictors of AKI. ROC analysis suggested that uNGAL1 had an excellent accuracy (0.97) and the higher sensitivity and specificity for predicting AKI (92.5 and 91.7%, respectively), with an optimal cutoff value of 13.2 ng/L and anticipating the diagnosis of AKI in 2.1 days.

Our results are similar to the AUC-ROC found in a study that followed children undergoing cardiopulmonary bypass and analyzed uNGAL and pNGAL as predictors of AKI, the concentration of uNGAL greater than 50 ??g/L predicted AKI at two hours following procedure in this population, with 100% sensibility and 98% specificity [7]. They were better than those found in pediatric septic patients by Wheeler et al. [15] and in adult septic patients in study performed by M°artensson et al, Royakkers et al and Si Nga et al [18, 38, 39]. The authors showed that the AUC was around 0.7 with sensitivity between 75 and 85% and specificity 35 and 40%.

Concerning uNGAL as predictor of death, in our study uNGAL and uNGAL/uCr were poor predictors (AUC ROC was lower than 0.7, sensitivity between 65 and 77% and specificity lower than 60%). We believe that adding any other marker, KIM-1, for example, with higher specificity, would help to improve the predictive value of the studied markers.

Few studies have shown an association between NGAL and mortality. Nickolas et al. [40] showed that uNGAL was associated with clinical outcomes, including consultation with nephrologist, dialysis, and ICU admission (OR = 24.71 (CI: 7.69 to 79.42). Collins et al. [41] evaluated 399 patients with acute cardiac dysfunction and found that uNGAL between 12 and 24 h after treatment initiation was predictive of 30-day mortality (?? = 0.02).

Finally, our results agree with the recent systematic review published in 2016 by An Zhang *et al* [40]. The authors analyzed 15 studies and 1478 patients and observed a precision of 0.86 for pNGAL and 0.9 for uNGAL, corroborating the importance of NGAL as a predictor of AKI associated with sepsis. Only one prospective observational study of 92 septic patients with AKI included in that meta-analysis evaluated the role of NGAL as biomarker for death, and it showed that APACHE 2 and uNGAL were independent predictors for mortality at 180 days, with OR of 0.81 (95% CI 0.72-0.90) and 0, 76 (95% CI: 0.66-0.86), respectively, both with *p* <0.05.

The present study has some important limitations. It included a small number of patients and was performed in single center. Due to the small number of patients, no analysis of uNGAL according to the stage of AKI was performed. The role of uNGAL as a predictor of dialysis also was not evaluated. Despite these limitations, it was the first study that results allow us to conclude that uNGAL is elevated in septic elderly patients but statistically higher in those with sepsis and AKI and reaffirm the role of uNGAL to predict AKI. uNGAL values within 2 after admission to ICU were excellent predictors of AKI in septic elderly patients, being highly sensitive and specific.
